# Replication and exploratory analysis of 24 candidate risk polymorphisms for neural tube defects

**DOI:** 10.1186/s12881-014-0102-9

**Published:** 2014-10-08

**Authors:** Faith Pangilinan, Anne M Molloy, James L Mills, James F Troendle, Anne Parle-McDermott, Denise M Kay, Marilyn L Browne, Emily C McGrath, Hatice Ozel Abaan, Marie Sutton, Peadar N Kirke, Michele Caggana, Barry Shane, John M Scott, Lawrence C Brody

**Affiliations:** Molecular Pathogenesis Section, Genome Technology Branch, National Human Genome Research Institute, National Institutes of Health, Room 5306, 50 South Drive, Bethesda, MD 20892-8004 USA; Department of Clinical Medicine, Trinity College Dublin, Dublin, Ireland; Division of Epidemiology, Statistics and Prevention Research, Eunice Kennedy Shriver National Institute of Child Health and Human Development, National Institutes of Health, Bethesda, MD USA; Office of Biostatistics Research, National Heart Lung and Blood Institute, National Institutes of Health, Bethesda, MD USA; School of Biotechnology, Dublin City University, Dublin, Ireland; New York State Department of Health, Division of Genetics, Wadsworth Center, Albany, NY 12201 USA; New York State Department of Health, Congenital Malformations Registry, Troy, NY 12180 USA; Department of Epidemiology and Biostatistics, School of Public Health, University at Albany, Rensselaer, NY 12144 USA; Evidence Centre, Health Research Board, Dublin, Ireland; Child Health Epidemiology Unit, Health Research Board, Dublin, Ireland; Department of Nutritional Sciences and Toxicology, University of California, Berkeley, CA 94720-3104 USA; School of Biochemistry and Immunology, Trinity College Dublin, Dublin, Ireland

**Keywords:** Neural tube defects, Spina bifida, Folate, Folic acid, One-carbon metabolism, Replication

## Abstract

**Background:**

Neural tube defects (NTDs), which are among the most common congenital malformations, are influenced by environmental and genetic factors. Low maternal folate is the strongest known contributing factor, making variants in genes in the folate metabolic pathway attractive candidates for NTD risk. Multiple studies have identified nominally significant allelic associations with NTDs. We tested whether associations detected in a large Irish cohort could be replicated in an independent population.

**Methods:**

Replication tests of 24 nominally significant NTD associations were performed in racially/ethnically matched populations. Family-based tests of fifteen nominally significant single nucleotide polymorphisms (SNPs) were repeated in a cohort of NTD trios (530 cases and their parents) from the United Kingdom, and case–control tests of nine nominally significant SNPs were repeated in a cohort (190 cases, 941 controls) from New York State (NYS). Secondary hypotheses involved evaluating the latter set of nine SNPs for NTD association using alternate case–control models and NTD groupings in white, African American and Hispanic cohorts from NYS.

**Results:**

Of the 24 SNPs tested for replication, *ADA* rs452159 and *MTR* rs10925260 were significantly associated with isolated NTDs. Of the secondary tests performed, *ARID1A* rs11247593 was associated with NTDs in whites, and *ALDH1A2* rs7169289 was associated with isolated NTDs in African Americans.

**Conclusions:**

We report a number of associations between SNP genotypes and neural tube defects. These associations were nominally significant before correction for multiple hypothesis testing. These corrections are highly conservative for association studies of untested hypotheses, and may be too conservative for replication studies. We therefore believe the true effect of these four nominally significant SNPs on NTD risk will be more definitively determined by further study in other populations, and eventual meta-analysis.

**Electronic supplementary material:**

The online version of this article (doi:10.1186/s12881-014-0102-9) contains supplementary material, which is available to authorized users.

## Background

Neural tube defects (NTDs) are common, severe birth defects that affect the development of the spinal cord and/or brain. NTDs mainly present as spina bifida, anencephaly and encephalocele. Although the NTD rate has been reduced in areas with folic acid fortification [1-4], reported NTD rates include ~1 in 1000 births in the US [5,6], 0.1-1.1 in 1000 births in Europe [7], 1–4.8 in 1000 pregnancies in China [3], 3.9-8.8 in 1000 births in India [8], and 0.2-9.6 in 1000 births in Latin America [9]. Both environmental and genetic factors are involved in the development of NTDs. In western countries, dietary folate is the strongest known environmental factor. Mothers with an NTD pregnancy have significantly lower levels of folate [10], and periconceptional folate supplementation can lower NTD risk by 50-70% [3,11,12]. The most firmly established genetic factor is a common polymorphism (rs1801133, c.677C > T) in the 5, 10-methylene-tetrahydrofolate reductase (*MTHFR*) gene. The alternate nucleotide in this single nucleotide polymorphism (SNP) changes an alanine to a valine (p.A222V), resulting in a thermolabile form of the protein [13]. The *MTHFR* c.677 TT genotype has been significantly associated with NTD cases in many populations (reviewed in [14,15]). Other NTD risk SNPs have been identified in a number of populations (e.g., rs2236225 (c.1958G > A, p.R653Q) in methylenetetrahydrofolate dehydrogenase (NADP + dependent) 1, methenyltetrahydrofolate cyclohydrolase, formyltetrahydrofolate synthetase (*MTHFD1*) [16-21], rs1805087 (c.2756A > G, p.D919G) in methionine synthase (*MTR*) [15,16,22-29], rs1051266 (c.80A > G, p.H27R) in the reduced folate carrier (*RFC1*/*SLC19A1*) [15,21,30-33]), and rs70991108 (19 bp indel; c.86 + 59_86 + 60insACCTGGGCGGGACGCGCCA) in dihydrofolate reductase (*DHFR*) [34-36]). The majority of associations have not been subjected to robust replication.

Rigorously establishing genetic risk for any multifactorial disorder is important but inherently difficult. Genetic risk is optimally detected with a large number of racially/ethnically homogenous samples that include cases with a well-defined, highly penetrant phenotype. These are difficult criteria to meet for rare, complex disorders, meaning that statistical power is often compromised to some extent. This, in combination with the bias that significant associations are more likely to be published [37-40], contributes to many associations in the literature that would not survive correction for multiple tests.

Specifically in the field of genetic risk for NTDs, studies including large numbers of candidate polymorphisms and/or genes have implicated many candidate SNPs exhibiting nominal NTD associations [21,41-45]. Each of the cited studies examined a minimum of 37 SNPs for NTD risk, yet only the association of rs1907362 in cubilin (*CUBN*) with NTDs survived correction in the original study [21]. The most likely explanation is that the reported nominally significant associations were due to chance. However, the large number of tests performed, reduced power due to limited availability of samples, and/or the population-specific effect of a risk allele can all contribute to Type II errors. Therefore replication studies are essential for the validation of any genetic risk factor for NTDs.

In one of the largest studies to date we previously reported evaluation of the common genetic variation in 82 candidate genes for NTD risk in an Irish population [45]. The inclusion of 570 NTD cases (~95% spina bifida cases), their parents and 999 controls made it possible to use both family based methods (the transmission disequilibrium test and log-linear analysis) and case–control based methods (logistic regression) to evaluate genetic risk for NTDs in cases (case effect) as well as genetic risk for an NTD pregnancy in mothers (maternal effect). In the current study, we test 24 of the resulting nominally significant association signals using 9 case–control tests and 14 family-based tests in racially/ethnically similar populations. The 9 SNPs that showed nominal significance by case–control analysis in the previously studied Irish NTD cohort were additionally evaluated for NTD risk using varying models in racially/ethnically similar and distinct populations.

## Methods

### Statistical methods

For replication analyses, each candidate risk allele was retested using the same association test and model for which it was nominally significant in the primary study. These tests are noted in Tables 1 and 2, and include: 1) case–control tests of logistic regression using a continuous, recessive or dominant genetic model; 2) log-linear tests of case or maternal effect using a dominant or recessive genetic model; and 3) the Spielman transmission disequilibrium test (TDT [46]).

**Table 1:** **Case–control replication analyses in the New York State NTD cohort**

**Gene SNP**	Test/Model*	**Result in Irish NTD Cohort [** 45 **]**	**Result in NYS NTD Cohort**
		**GRR [95% CI], p-value**	**GRR [95% CI], p-value**
*ADA* rs6031682	LR CC Dom	0.67 [0.52-0.86], p = 0.0016	0.92 [0.63-1.33], p = 0.653
*ALDH1A2* rs7169289	LR CC Dom	0.71 [0.56-0.90], p = 0.0043	1.07 [0.77-1.47], p = 0.697
*ARID1A* rs11247593	LR CC Cont	1.35 [1.11-1.64], p = 0.0027	0.81 [0.63-1.04], p = 0.092
*CDKN2A* rs7041637	LR CC Cont	0.69 [0.53-0.89], p = 0.0048	1.07 [0.85-1.36], p = 0.564
*CTH* rs12733999	LR CC Rec	0.57 [0.39-0.84], p = 0.0048	1.12 [0.65-1.92], p = 0.692
*ENOSF1* rs1059384	LR CC Rec	1.84 [1.23-2.75], p = 0.0028	0.98 [0.60-1.60], p = 0.937
*FOLH1* rs383028	LR CC Cont	0.58 [0.39-0.85], p = 0.0051	1.07 [0.76-1.52], p = 0.697
*MFTC* rs17803441	LR CC Cont	0.62 [0.48-0.81], p = 0.0003	1.02 [0.66-1.58], p = 0.922
*MTHFD1L* rs2295083	LR CC Rec	0.60 [0.43-0.85], p = 0.0038	1.25 [0.89-1.76], p = 0.193

*LR CC = logistic regression, case–control; genetic models: Dom (dominant), Cont (continuous) and Rec (recessive).

**Table 2:** **Family-based replication analyses in the combined United Kingdom NTD cohort**

**Gene SNP**	Test/Model*	**Result in Irish NTD Cohort [** 45 **]**	**Result in UK Cohort**
		**GRR [95% CI], p-value**	**GRR [95% CI], p-value**
*ADA* rs452159	LL Case Dom	2.28 [1.29-4.05], p = 0.0047	1.86 [1.01-3.40], p = 0.045
*CDKN2A* rs3218009	LL Mat Dom	2.32 [1.45-3.71], p = 0.0004	1.16 [0.81-1.65], p = 0.418
*COMT* rs174675	LL Case Dom	0.41 [0.23-0.73], p = 0.0028	0.89 [0.55-1.43], p = 0.621
*CUBN* rs11254375	LL Mat Dom	1.48 [1.10-1.99], p = 0.0097	1.22 [0.89-1.66], p = 0.210
*DNMT3A* rs7560488	LL Case Rec	2.10 [1.31-3.38], p = 0.0021	0.89 [0.61-1.28], p = 0.520
*DNMT3B* rs6058896	TDT	1.88 [1.24-2.85], p = 0.0029	1.18 [0.77-1.82], p = 0.443
*FOLH1* rs16906205**	LL Mat Dom	0.43 [0.23-0.78], p = 0.0060	1.10 [0.75-1.63], p = 0.619
*GART* rs2070388	TDT	0.53 [0.36-0.78], p = 0.0012	0.73 [0.52-1.03], p = 0.070
*GNMT* rs9462856	LL Case Dom	0.55 [0.36-0.83], p = 0.0048	1.19 [0.85-1.68], p = 0.308
*MAT2B* rs17535909	LL Case Rec	1.69 [1.18-2.42], p = 0.0045	Failed genotyping
*MTHFD1* rs11627525	LL Mat Rec	2.17 [1.24-3.79], p = 0.0067	1.13 [0.76-1.68], p = 0.545
*MTHFD1* rs2236225	LL Case Dom	1.66 [1.16-2.37], p = 0.0054	1.21 [0.84-1.74], p = 0.299
*MTR* rs10925260	LL Case Rec	0.64 [0.48-0.86], p = 0.0031	0.71 [0.53-0.94], p = 0.019
*PEMT* rs4646402	LL Case Dom	0.600 [0.46-0.8], p = 0.0005	0.92 [0.70-1.23], p = 0.585
*PEMT* rs16961845	LL Mat Rec	1.92 [1.18-3.11], p = 0.0082	0.73 [0.46-1.15], p = 0.171

*LL; = log-linear analysis; TDT = transmission disequilibium test; Case = case effect; Mat = maternal effect; genetic models: Dom (dominant) and Rec (recessive).

**Proxy SNP (*FOLH1* rs11040291; r^2^ = 1 in Hapmap CEU) results shown for replication.

Secondary hypotheses involved more broadly testing the candidate SNPs for NTD risk in other populations and/or NTD classifications. Three models (continuous, recessive and dominant) of logistic regression were performed to generate genotype relative risks (GRRs) and 95% confidence intervals (CI).

### Study populations

Family-based replication was performed in a United Kingdom cohort consisting of trios of NTD cases and their parents. Exclusion of the “Other NTDs” class, which may include a small number of families with multiple defects or spina bifida occulta, did not substantially change the results. This cohort consists of three groups (Table 3). First, the 400 case families recruited with the assistance of the UK Association for Spina Bifida and Hydrocephalus (ASBAH) (England and Wales) are fully described elsewhere [47]. Second, 131 case families were recruited from Northern Ireland. Ethical approval of the use of these samples was granted by the UK Multi-Centre Research Ethics Committee (University of Newcastle, UK, and University of Northern Ireland, Coleraine) and the Institutional Review Board at the National Human Genome Research Institute (Bethesda, MD, USA).

**Table 3:** **Number of NTD family members in the UK family-based cohort**

	**England**	**Northern Ireland**	**Wales**	**Total**
Spina bifida	331	122	26	479
Encephalocele	7	5	1	13
Spina bifida and Encephalocele	2	0	0	2
Other NTDs*	28	4	5	37
Total NTD Cases	368	131	32	531
NTD Mothers	321	121	30	472
NTD Fathers	263	105	21	389

*May include NTD in the context of multiple defects or spina bifida occulta.

Replication of findings from case–control analyses was performed in a NYS case–control cohort. NTD cases from the entire state born 1998–2005 were identified by their inclusion in the NYS Congenital Malformations Registry (NYSCMR). Matched controls were selected as a random sample of non-malformed control infants born 1998–2005 from NYS Newborn Screening Program records. Demographic data was obtained by matching to NYS birth certificates. Four controls matched for maternal race/ethnicity were selected for each NTD case. Due to low numbers, the Asian and “other” racial/ethnic subgroups were excluded, and subjects coded as “Hispanic, white” and “Hispanic, other” were combined into a single Hispanic group for analysis. Case diagnoses allowed classification of the NTDs into six subgroups based on NTD subtype (anencephaly, encephalocele, spina bifida) and whether the NTD was isolated or part of multiple defects (Table 4). To perform the replication analyses, only isolated NTDs of all three subtypes among the NHWs were considered in order to most closely match the composition of the NTD cases in the original Irish cohort. Secondary hypotheses involved analyses of: 1) all NTD cases and all controls, stratified by race/ethnicity; and 2) isolated spina bifida cases and corresponding matched controls. Approval for the use of the de-identified samples was granted by the New York State Department of Health Institutional Review Board and the NIH Office for Human Research Protections.

**Table 4:** **Number of NTD cases and controls by race/ethnicity in the NY state cohort**

	**White, non-Hispanic**	**AA, non-Hispanic**	**Hispanic, white**	**Hispanic, other**	Asian*	Other/Unknown*	**Total**
Isolated anencephaly	7	2	1	1	0	0	11
Isolated encephalocele	22	10	12	5	1	1	51
Isolated spina bifida	161	65	65	13	16	2	322
Anencephaly - multiple defects	2	3	1	0	1	0	7
Encephalocele - multiple defects	16	5	4	1	0	0	26
Spina bifida - multiple defects	25	13	11	1	2	0	52
Total NTD cases	233	98	94	21	20	3	469
Controls	941	408	393	87	91	12	1932

*Excluded from analysis due to low numbers.

### Genotyping

*MTHFD1* rs2236225 was genotyped in the UK cohort as a restriction fragment length polymorphism (PCR-RFLP) using *Msp*I as previously described [17,48]. Genotypes were obtained for 91.6% of NTD fathers, 92.5% of NTD cases and 93.6% of NTD mothers. Concordance was 100% for repeated (n = 83) and for re-plated (the testing of a second sample from the DNA source) (n = 92) samples using an independent assay based on detection of allele-specific primer extension using matrix-assisted laser desorption/ionization – time of flight (MALDI-TOF) mass spectrometry (Sequenom, San Diego, CA, USA). The remaining SNPs in Table 2 were also genotyped in the UK cohort using the Sequenom platform. Two independent assays of folate hydrolase (*FOLH1*) rs16906205 failed, so the proxy *FOLH1* rs11040291 (r^2^ = 1 in Hapmap CEU) was genotyped and reported instead. Two independent assays to genotype methionine adenosyltransferase II, beta (*MAT2B*) rs17535909, were attempted but both failed, and there was no proxy for this singleton SNP. For this set of 13 SNPs, the average call rates were ≥96.8% for each family group (NTD cases, NTD mothers or NTD fathers). Re-plated and re-genotyped samples covered >18% of the cohort with 99.2% genotype concordance for this set of 13 SNPs. The 14 SNPs typed in the UK cohort exhibited non-Mendelian inheritance in <1% of families. These SNPs were also in Hardy Weinberg Equilibrium (HWE, p > 0.01) for each family group. Genotypes for families exhibiting non-Mendelian inheritance and other discordant genotypes were excluded from analysis.

For NYS samples, DNA was extracted from one 3-mm archived dried blood spot specimen [49] and whole-genome amplified using a primer extension pre-amplification method, as described previously [50]. SNPs were genotyped by KBiosciences (Herts, UK) using KASPar chemistry. Eight SNPs were genotyped in duplicate using independently whole-genome amplified DNA aliquots with 100% concordance in genotype calls. *FOLH1* rs383028 was genotyped using genomic DNA because data from amplified DNA did not pass quality control criteria. The average call rate for 9 SNPs (Table 1) was 99.9% for both cases and controls. Replated samples covered 6.5% of the cohort with genotype concordance of 100%. No SNPs deviated from HWE (p > 0.01) in any case or control group for any race/ethnicity.

## Results

The primary aim of this study was to perform replication analyses of the nominally significant NTD associations identified in a recent study in an Irish population [45]. The secondary aim was to test a subset of these candidate SNPs for association using alternate risk models and populations.

### Replication analyses

#### Replication criteria

The replication strategy was designed to retest nominally significant NTD-associated SNPs in racially/ethnically matched populations using the same association tests and genetic models that previously yielded the lowest p-values among 1441 SNPs in 82 candidate genes tested in an Irish population [45]. Case–control association tests were performed in a cohort of 190 isolated NTD cases and 941 controls from non-Hispanic white (NHW) mothers from NYS, and family-based tests were performed in NTD trios (n = 530) consisting of NTD cases and their parents from the United Kingdom, including centers in Northern Ireland, England and Wales.

The top 25 groups of SNPs sharing high linkage disequilibrium (LD; D’ > 0.9) with the lowest p-values for any test were selected for replication (52 SNPs total). Without access to NTD mothers and corresponding controls several loci could not be retested. This included 9 independent mother-control signals (17 SNPs) in adenosine deaminase (*ADA*), alcohol dehydrogenase 1 family, member A2 (*ALDH1A2*), catechol-O-methyltransferase (*COMT*), *CUBN*, *MTHFD1,* methylenetetrahydrofolate dehydrogenase (NADP + dependent) 1-like (*MTHFD1L*), brachyury (*T*), and transcription factor AP-2 alpha (*TFAP2A*). Of the remaining 35 SNPs, the SNP with the lowest observed p-value was selected for replication testing whenever SNPs with a minimum p-value for the same test and model shared high LD (D’ > 0.9). This reduced the number of tests to 24 (9 case–control tests and 15 family-based tests). Results of these tests are shown in Tables 1 and 2.

#### Replication of associations detected in case–control analyses

We used the NTD case samples from NYS to replicate case effects previously observed in the Irish NTD cohort. Each SNP was tested for association with NTDs by logistic regression with the same genetic model used for the original observation. No SNP was observed to be significantly associated with isolated NTDs in white cases from NYS (Table 1). This lack of replication was accompanied by the corresponding genotype relative risk (GRR) values indicating an inconsistent effect of the candidate risk allele to that observed in the original study for eight of the nine SNPs.

#### Replication of associations detected in family-based analyses

Replication analyses of previously observed case or maternal effects detected by log-linear analysis were repeated with the same genetic model in a combined cohort of NTD triads from the United Kingdom. Two SNPs showed nominally significant association with NTDs: *ADA* rs452159 in a dominant model (GRR = 1.86 [1.01-3.40], p = 0.045), and *MTR* rs10925260 in a recessive model (GRR = 0.71 [0.53-0.94], p = 0.019). In both cases, the magnitude of the effect and the specific risk allele was similar to the original observation in the Irish NTD cohort (Table 2). If applied, the significance of these results would not withstand Bonferroni correction for multiple tests when considering all SNPs tested in the current study. In contrast to the case–control analyses, the direction of effect for the GRRs of these family-based associations largely agreed between the initial and replication studies (10 of 14), regardless of significance.

### Secondary hypotheses - exploratory analyses in new populations

#### Applying analyses that yielded nominal associations from the initial study

In addition to replication, these candidate SNPs were tested for association in other NTD populations using various models. The nine SNPs selected for case–control replication in the NYS cohort were first tested in African American and Hispanic cases with an isolated NTD and controls using the same tests and models for which each SNP had been originally observed to be nominally associated in the Irish population (Table 1, 2 tests/SNP). Of the nine SNPs examined in each of the two racial/ethnic groups, only one, *ALDH1A2* rs7169289, was found to be nominally associated with isolated NTDs -- in African Americans in a continuous model (GRR = 0.57 [0.34-0.98], p = 0.041). This “protective” effect is in contrast to the risk effect seen for this SNP in the Irish cohort using a dominant model.

#### Case–control analyses in all NTD cases vs. isolated Spina Bifida cases

These SNPs were also tested by performing logistic regression using three genetic models (continuous, dominant and recessive) in all NTD cases and controls in each of the three racial/ethnic groups (9 tests/SNP). Of these nine SNPs tested in nine models (N = 81 tests), AT rich interactive domain 1A (*ARID1A*) rs11247593 was the only SNP found to be nominally associated with NTDs -- in non-Hispanic whites in a dominant model (GRR = 0.58 [0.35-0.97], p = 0.037). This protective effect is in contrast to the risk effect (GRR > 1) seen in the Irish cohort using a continuous model (Table 1).

Lastly, the same tests were performed in a restricted subset of isolated spina bifida cases and controls in the three racial/ethnic groups (9 tests/SNP). These 81 tests generated two significant findings: *ALDH1A2* rs7169289 was nominally associated with isolated spina bifida cases in the African American population in continuous (GRR = 0.46 [0.25-0.87], p = 0.017) and dominant (GRR = 0.47 [0.24-0.94], p = 0.033) models. Similarly, a significant risk effect was observed in the original Irish population, but not the NYS NHW population (Table 1).

These results are summarized in Table 5. These results are nominally significant, though would not withstand Bonferroni correction using the total number of tests as the correction factor.

**Table 5:** **Nominally significant associations in the New York state cohort - secondary hypotheses**

**Gene SNP**	**Race/ Ethnicity**	**Freq Risk Allele**	**NTD**	**Test/Model**	**GRR [95% CI], p-value**
*ALDH1A2* rs7169289	AA	0.17	Isolated NTDs	LR Cont	0.57 [0.34-0.98], p = 0.041
*ARID1A* rs11247593	NHW	0.75	All NTDs	LR Dom	0.58 [0.35-0.97], p = 0.037
*ALDH1A2* rs7169289	AA	0.17	Isolated SB	LR Cont	0.46 [0.25-0.87], p = 0.017
*ALDH1A2* rs7169289	AA	0.17	Isolated SB	LR Dom	0.47 [0.24-0.94], p = 0.033

## Discussion

Our study addressed two questions. First, are SNPs that were nominally significant upon testing for NTD association in an Irish population also associated with NTDs in a similar, independent population? Second, are the SNPs that were nominally significant upon testing for NTD association in an Irish population by logistic regression also associated with NTDs in different racial/ethnic populations when using a broader range of association tests, genetic models and NTD case groupings?

Our criteria for replication were stringent, and only performed for a SNP when the same association test and genetic model could be applied. Because we did not have samples from mothers of NYS NTD cases, we were unable to test for a maternal risk for nine independent mother-control signals in seven genes. Nine SNPs were re-tested for a case effect by logistic regression in a white NTD sample from NYS, and log-linear analyses were used to re-test ten SNPs for a case effect and five SNPs for a maternal effect in a UK NTD sample. Of 14 SNPs previously observed to be associated with NTDs by family-based analysis in an Irish population, two showed nominally significant NTD association in trios from the UK (Table 2). *ADA* rs452159 falls in the first intron, and is at the border of a D’ block encompassing the first exon and intron of the gene. *MTR* rs10925260 is in intron 23 of the gene, and is part of a large block of D’ LD encompassing the entire gene. Due to their strong D’ linkage, these nominally significant associations may reflect a direct signal or that of a causative SNP linked to the tested SNP(s).

Although none of the 9 SNPs found to be nominally significant in Irish NTDs were replicated in isolated NYS white NTD cases, two were nominally associated with NTDs under different conditions. *ARID1A* rs11247593 did not replicate by logistic regression using a continuous model in isolated NTDs (Table 1), but was significant when all white NTD cases and controls were tested using a dominant model (Table 5). *ARID1A* rs11247593 is intronic and is part of a large D’ block extending over the entire gene, so the signal may be due to the tested SNP or a linked causative SNP. In addition, *ALDH1A2* rs7169289 was significantly associated with isolated NTDs and isolated spina bifida cases in the African American population (Table 5). These results most likely represent a single association signal as the genetic models (continuous, dominant) and NTD sets (isolated NTDs, isolated SB) for these analyses overlap. The estimated effects observed in African American NTD cases from NYS (Table 5; OR = 0.46-0.57, p = 0.017-0.041) are similar to the original effect observed in an Irish population (Table 1; OR = 0.67 [0.52-0.86], p = 0.0016). *ALDH1A2* rs7169289 is just downstream of the gene. It falls between two blocks of D’ LD, and the association signal may be due to this SNP or a linked SNP in either block.

As was found in the original study, none of the observed associations in the NYS and UK sample sets would have survived correction for multiple tests. One interpretation is that these associations are indeed due to chance and that the tested SNPs do not contribute to NTD risk. It is not clear, however, that Bonferroni correction is appropriate for replication studies. All the tests performed in this study were clearly based on individual, *a priori* hypotheses generated for our initial study and supported by previous data. Additionally, factors that can contribute to Type II error should be considered, such as population stratification and genetic heterogeneity.

The most important factor may be limited sample size, which compromises the power to detect true associations. Compared to the number of NTD cases (n ~ 570) in the original Irish cohort, a limited number of white NYS isolated NTD cases (n ~ 190) were available in the current study. With ~530 NTD trios, however, the UK NTD cohort appears comparable, but this is before taking into consideration the power required to replicate an association. Due to the “winner’s curse,” or the tendency of an initial observation to overestimate the effect size or significance of a true association, replication studies generally require larger study sample numbers to detect the original effect [51,52]. This may explain why the well known NTD risk allele of *MTHFR* rs1801133 (T) is not associated with NTDs by TDT (p = 0.742) in our large UK NTD cohort. In fact, it is nominally significant as a protective factor in a recessive model by log-linear analysis (GRR = 0.663 [0.441-0.997], p = 0.049). Considering that this association has been replicated in many studies in other populations supporting its role in NTD risk, it would be surprising if the MTHFR rs1801133 TT genotype does not contribute to NTDs in the UK population. A lack of replication power and chance seem the most likely explanations for this failure to replicate, which must be taken into consideration for the other SNPs tested for replication in this study. Authentic validation of any genetic association requires accumulation of evidence over time, involving multiple studies in independent populations.

Lack of adequate power is a pervasive problem in the field of NTD genetics. One recent review estimated that ~1000 cases would be required to attain 80% power to detect an odds ratio of 2 or under, yet approximately one quarter of published NTD association studies used fewer than 100 cases [53]. This problem is compounded when attempting to evaluate candidate risk SNPs in less studied populations. While we were able to examine candidate risk SNPs of interest in African American and Hispanic populations from NYS, far fewer cases were available compared to whites (Table 4). Although some population-based studies have included African American cases in aggregate analyses of genetic risk for NTDs [54-56], and there are studies of NTD risk in African cohorts [57], this is the first report of candidate NTD SNPs evaluated in an African American case–control cohort. The nominal association observed for *ALDH1A2* rs7169289 may be real, but needs confirmation.

Identifying potential NTD risk SNPs has proven much easier than validating them. A single replication study is not definitive, and the limited numbers in existing NTD cohorts may contribute to subsequent underpowered studies failing to confirm reported associations. By combining data from multiple published studies, meta-analyses increase power and confidence in whether a SNP truly contributes to NTD risk. Meta-analyses involving aggregate data from several hundred to several thousand NTD cases and controls have confirmed *MTHFR* rs1801133 (c.677C > T) as a maternal [58] and case risk factor for NTDs [14,15,58,59]. Although comparatively fewer data were available, meta-analyses show 5-methyltetrahydrofolate-homocysteine methyltransferase reductase (*MTRR*) rs1801394 (c/66A > G) is a maternal risk factor for NTDs [60,61], while *MTR* rs1805087 (c/2756A > G) does not contribute to maternal or case risk [15,22,60,62]. However, meta-analysis requires a large amount of data including full genotype information. These data are absent in publications for the majority of NTD risk SNPs that show nominal significance. It is therefore essential to publish all association study data in a format that allows future meta-analyses to be performed (i.e., genotype counts or a way to unambiguously determine them). As such, we have reported the genotype data for all 24 SNPs tested in our study (Additional file 1: Table S1 and Additional file 2: Table S2).

## Conclusions

In summary, this study represents both the effort to validate nominally significant NTD associations via replication, and to test a subset of these SNPs in alternate NTD groupings and racial/ethnic populations. The NTD associations we observed with four SNPs would not have survived correction for multiple tests. However, we believe it would be overly severe to apply Bonferroni correction to *ADA* rs452159 and *MTR* rs10925260, which were nominally significant by strict replication analysis, as well as *ARID1A* rs11247593, which was found to be associated by a different model and expanded definition of NTDs. Additionally, to our knowledge, this is the first report of a genetic association with NTDs in African Americans and so the nominal significance of *ALDH1A2* rs7169289 in the small NYS NTD cohort warrants follow-up. Further testing in independent populations and meta-analyses are needed to clarify the role of these and a multitude of nominally significant SNPs associated with NTDs.

## Additional files

Additional file 1: Table S1. Genotype counts for candidate SNPs in the NTD cases, fathers and mothers from the United Kingdom (England, Wales, and Northern Ireland). Additional file 2: Table S2. Genotype counts for candidate SNPs in the New York state NTD cases and controls by ethnicity and each analyzed NTD subtype.

## Abbreviations

LD: Linkage disequilibrium
NTDs: Neural tube defects
SNP: Single nucleotide polymorphism
TDT: Transmission disequilibrium test
NYS: New York State
NHW: Non-Hispanic white
GRR: Genotype relative risk
